# Rumen Virus Populations: Technological Advances Enhancing Current Understanding

**DOI:** 10.3389/fmicb.2020.00450

**Published:** 2020-03-26

**Authors:** Rosalind A. Gilbert, Eleanor M. Townsend, Kathleen S. Crew, Thomas C. A. Hitch, Jessica C. A. Friedersdorff, Christopher J. Creevey, Phillip B. Pope, Diane Ouwerkerk, Eleanor Jameson

**Affiliations:** ^1^Department of Agriculture and Fisheries, Brisbane, QLD, Australia; ^2^Queensland Alliance for Agriculture and Food Innovation, The University of Queensland, St Lucia, QLD, Australia; ^3^Warwick Integrative Synthetic Biology Centre, School of Life Sciences, University of Warwick, Coventry, United Kingdom; ^4^Functional Microbiome Research Group, Institute of Medical Microbiology, RWTH University Hospital, Aachen, Germany; ^5^Institute of Biological, Environmental and Rural Sciences, Aberystwyth University, Aberystwyth, United Kingdom; ^6^Institute for Global Food Security, School of Biological Sciences, Queen’s University Belfast, Belfast, United Kingdom; ^7^Faculty of Chemistry, Biotechnology and Food Science, Norwegian University of Life Sciences, Ås, Norway; ^8^Faculty of Biosciences, Norwegian University of Life Sciences, Ås, Norway

**Keywords:** rumen, virus, phage, metagenomics, proteomics, biofilm, methanogen

## Abstract

The rumen contains a multi-kingdom, commensal microbiome, including protozoa, bacteria, archaea, fungi and viruses, which enables ruminant herbivores to ferment and utilize plant feedstuffs that would be otherwise indigestible. Within the rumen, virus populations are diverse and highly abundant, often out-numbering the microbial populations that they both predate on and co-exist with. To date the research effort devoted to understanding rumen-associated viral populations has been considerably less than that given to the other microbial populations, yet their contribution to maintaining microbial population balance, intra-ruminal microbial lysis, fiber breakdown, nutrient cycling and genetic transfer may be highly significant. This review follows the technological advances which have contributed to our current understanding of rumen viruses and drawing on knowledge from other environmental and animal-associated microbiomes, describes the known and potential roles and impacts viruses have on rumen function and speculates on the future directions of rumen viral research.

## Introduction

The presence of viruses in the rumen of domesticated herbivores was first noted in the 1960s (Adams et al., 1966; Hoogenraad et al., 1967) and it was quickly recognized that these viruses were not just those ingested by chance from feed and water. Instead, the majority of viruses in the rumen are those which either co-exist, or actively replicate and predate on, the dense microbial populations of the rumen. While viruses are also thought to infect the eukaryote populations of the rumen (protozoa and fungi), the viruses infecting rumen bacteria, often referred to as bacteriophage or phages, are the most well characterized. More recently, with the increasing focus on enteric methane emissions and rumen methanogenesis, viruses infecting methanogenic archaea (archaeal viruses or archaeaphage) have also been investigated.

Viruses of rumen microbes are an important focus for study, both as a key player in our understanding of rumen microbial community function and as a potential alternative to antibiotics in the face of a potential post-antibiotic era, as either a prophylaxis or treatment for problematic microbes. This review aims to summarize current knowledge of rumen virus populations, encompassing the traditional methods used to study rumen virus populations, as well as new and emerging technologies. We also draw on findings from other, better studied, microbial ecosystems (other animals, water, soils and microbial biofilms) to provide a fresh perspective and improve our understanding of the roles viral populations have in the rumen microbiome.

## Technologies for Studying Rumen Virus Populations

Developments in rumen virus research have been closely aligned with the technologies available for their study (Table 1). While the existence of viruses infecting bacteria were first noted in the early 1900’s (Twort, 1915; d’Herelle, 1918), it was the development of electron microscopy (EM) in the 1930’s (Haguenau et al., 2003) that showed that phages were actually virus particles and enabled rumen viral populations to be observed and enumerated (Hoogenraad et al., 1967; Paynter et al., 1969). Advances in methodology for the *in vitro* culture of rumen microbes (Hungate, 1969) enabled the cultivation of obligately anaerobic bacteria from the rumen and consequently the isolation of virus particles and determination of their biological characteristics. The advent of molecular biology enabled the preliminary genetic characterization of virus isolates (Klieve et al., 1991) providing an alternative method to TEM for virus particle enumeration and the determination of virus population dynamics (Klieve and Swain, 1993).

**TABLE 1:** Applications, benefits and limitations of technologies used for the study of rumen viruses.

Method		Application and benefits	Limitations	Example publications
Microscopy	TEM	Visualization of viral particle morphology	Specialized equipment	Hoogenraad et al., 1967; Ritchie et al., 1970; Klieve and Bauchop, 1988
		Estimation of viral numbers	Sample preparation can bias enumeration	
			Time consuming and expensive	
			Cannot determine viral particle viability and biological attributes	
Molecular Biology	PFGE	Provides snapshot of viral community and estimation of viral numbers	Cannot provide taxonomic and functional gene information	Klieve and Swain, 1993; Swain et al., 1996a
Isolation		Confirms viral particle viability	Requires availability of susceptible microbial host	Klieve, 2005; Gilbert and Klieve, 2015
		Allows viral cultivation and storage in reference collections	Bias from sample preparation methods (e.g. exclusion of large viral particles)	
		Enables determination of biological parameters (host range, growth, replication and survival)		
		Allows extraction and sequencing of virus-specific nucleic acids		
		Allows viral protein purification		
Sequencing	Viral fraction	Provides snapshot of viral community structure	Requires isolation of intact viral particles from environmental samples	Breitbart et al., 2002; Clokie et al., 2011; Berg Miller et al., 2012; Anderson et al., 2017
		Provides taxonomic and functional gene information	Bias from sample preparation methods (e.g. exclusion of large viral particles)	
		Overcomes technical limitations from low concentrations of viral DNA in environmental samples	Bias from any DNA amplification steps	
			High percentage of uncharacterized viral genes limits annotation of gene function and viral taxonomy	
			Sequence assembly bias and challenges	
			Cannot determine viral particle viability and biological attributes	
			Difficult to identify viral lifecycles (e.g. lysogeny) and virus:host interactions	
	Metagenomics	Provides snapshot of viral community structure	Virus sequence numbers relatively low	Dinsdale et al., 2008; Willner et al., 2009
		Captures sequences from intact viral particles and integrated prophages	High percentage of uncharacterized viral genes limits annotation of gene function and viral taxonomy	
		Provides viral taxonomic and functional gene information	Difficult to identify viral lifecycles (e.g. lysogeny) and virus:host interactions	
			Cannot determine viral particle viability and biological attributes	
			Bias toward detection of double stranded DNA phages	
	Transcriptomics	Identifies actively replicating viral genes	Virus sequence numbers relatively low	Hitch et al., 2019
		Allows detection of viruses with RNA genomes	Bias toward identification of over-expressed viral genes (e.g. structural proteins)	
			High percentage of uncharacterized viral genes limits annotation of gene function and viral taxonomy	
			Difficult to identify viral lifecycles (e.g. lysogeny) and virus:host interactions	
			Cannot determine viral particle viability and biological attributes	
	Whole genome sequencing	Provides complete viral genome sequences	For lytic viruses requires pure, viable virus isolates	Leahy et al., 2010; Kelly et al., 2014; Gilbert et al., 2017
		Viral reference sequences increase the accuracy of sequence analysis	For lysogenic viruses requires viable prokaryote hosts containing intact, integrated prophage/s	
		Provides structural and functional viral protein information	High percentage of uncharacterized viral genes limits annotation of gene function	
		Indicates mechanisms of virus:host interactions and viral replication		
		Enables assignment of taxonomy and phylogenetic comparison		
Proteomics		Detects proteins produced by actively replicating viruses	Virus proteins found in relatively low concentrations	Solden et al., 2018
			Difficult to identify viral lifecycles (e.g. lysogeny) and virus:host interactions	
			High percentage of uncharacterized viral proteins limits functional annotation	
			Bias toward identification of over-produced viral proteins (e.g. structural proteins)	

In the last 10 years, advances in DNA sequencing has led to the more rapid and accurate detection of viral sequences in prokaryote genome sequences and the sequencing of complete phage genomes (Leahy et al., 2010; Gilbert et al., 2017). A combination of high-throughput DNA sequencing and bioinformatic analysis has facilitated viral metagenomics: the culture-independent analysis of all the viral genetic material in a rumen sample. This has allowed the taxonomic classification of viral populations and determination of the relative abundance of individual viral taxonomic groups (Berg Miller et al., 2012). Sequence analysis of rumen sample RNA (metatranscriptomics) has also been used to identify RNA virus populations (Hitch et al., 2019). In addition, alternative technologies are being applied for the isolation of virus particles (for example, chromatography) and enabling the expression of novel viral proteins *in vitro* (Figura et al., 2011; Krajacic et al., 2017; Altermann et al., 2018). The emerging field of proteomics, is also providing fresh insights into the roles viruses and the proteins produced by viruses, may play within the rumen microbial ecosystem (Solden et al., 2018).

### Transmission Electron Microscopy

Electron microscopy is a large research field, with applications in materials science as well as biology. The combination of electron microscopy and specialized staining techniques, enables the visualization of virus particle structures at nanometer scale, which could not otherwise be distinguished using conventional light microscopy. Several different electron microscopy technologies exist including scanning electron microscopy (SEM), transmission electron microscopy (TEM), high voltage electron microscopy, immunoelectron microscopy (IEM) and cryoelectron microscopy (cryoEM) with or without shadowing and three dimensional image reconstruction (reviewed by Ackermann, 2012; Romero-Brey and Bartenschlager, 2015). Of these technologies, TEM is the most widely used technique for the visualization of all types of viruses (Ackermann and Prangishvili, 2012; Popov et al., 2019).

The first use of TEM to visualize virus particles within rumen fluid, was published in 1967 (Hoogenraad et al., 1967). This study initially intended to determine the fate of bacterial cell walls within the rumen of sheep and involved direct examination of rumen samples by TEM. The rumen fluid was filtered through muslin and stained for negative contrast using potassium phosphotungstate. This methodology, however, revealed the presence of large numbers of free virus particles (icosahedral particles and tailed phages) and as the microbes were not removed prior to TEM, the attachment of phage particles to intact bacterial cells, including cocci and spirochetes, was also observed. This study was therefore pioneering not only in its use of the relatively new technique of TEM, but also in its hypothesis of what we now know to be true, that “phage may be a constant feature of the bacterial population of the rumen” (Hoogenraad et al., 1967).

Other studies then followed, employing TEM to conduct morphological surveys of rumen fluid collected from various ruminant species, including sheep, cattle and reindeer (Paynter et al., 1969; Ritchie et al., 1970; Tarakanov, 1972; Klieve and Bauchop, 1988). These surveys sought to enumerate the numbers of different viral morphotypes present, with results ranging from 6 (Paynter et al., 1969), 26 (Klieve and Bauchop, 1988), and more than 40 distinct morphotypes (Ritchie et al., 1970). In all the species of ruminants examined, the most abundant viral morphotypes were those with polyhedral heads and tubular tails, which are now classified within the viral order of tailed phages infecting bacteria, the Caudovirales. This viral order currently contains five families, the Myoviridae, Siphoviridae, Podoviridae, Ackermannviridae, and Herelleviridae, the last two being recently added (Adriaenssens et al., 2012; Barylski et al., 2020a, b). Of these, the short non-contractile tailed phages (family Podoviridae), long contractile-tailed phages (family Myoviridae) and many long non-contractile tailed phages (family Siphoviridae) were observed. These early surveys also sought to quantitate the size of the rumen viral populations using TEM to count the numbers of particles present. While counts varied, possibly as a consequences of differences in animal species as well as differences in sample preparation and counting methodology, virus particle counts ranged from 5 × 10^7^ particles per mL bovine rumen fluid (Paynter et al., 1969) to greater than 10^9^ particles per mL bovine rumen fluid (Ritchie et al., 1970). It was also noted that the numbers of phages in crude rumen fluid could exceed bacterial numbers ca. 2 to 10:1 (Ritchie et al., 1970).

**FIGURE 1:**
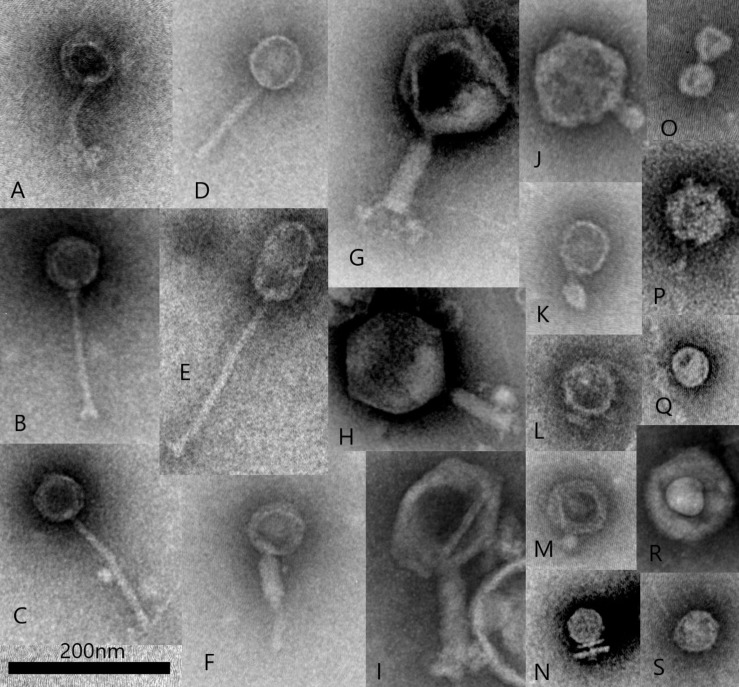
Transmission electron microscopy illustrating examples of common rumen virus morphotypes including Siphoviridae **(A–E)**, Myoviridae **(F–I)**, Podoviridae **(J–O)** and other virus-like-particles including potentially non-tailed phages **(P–S)**, such as Corticoviridae and Microviridae (scale bar 200 nm). All virus particles were purified from a single sample collected from an *in vitro* fermentation system (Infors) started with bovine rumen fluid. Virus particles were fixed with 2.5% glutaraldehyde and stained with 1% ammonium molybdate pH 7.0 and viewed with a JEOL JEM1400 Transmission Electron Microscope. Micrographs were captured using a Gatan Orius CCD camera.

Images from a more recent TEM morphological survey of a sample obtained from an anaerobic fermentation system started with an inoculum of bovine rumen fluid (Figure 1) shows virus particle types representative of those observed in previous studies. This survey also includes examples of non-tailed viral particles and while it can sometimes be difficult to distinguish the small tail structures of the Podoviridae, these particles could belong to other viral families infecting bacteria such as Tectiviridae, Corticoviridae, and Microviridae (Ackermann and Prangishvili, 2012). It is now known that morphological surveys may have technical limitations and operator bias can result in errors in identification (Ackermann, 2013). For example, tail-less virus-like-particles may be confused with tailed phages which have lost their tail structures either naturally, with some phages being more susceptible to tail loss in the environment than others, or as a result of particle concentration and TEM sample preparation (Williamson et al., 2012). In addition, filamentous phages (Inoviridae) can be associated with rumen bacteria (Klieve et al., 1989) and have been observed by morphological survey (Klieve and Bauchop, 1988), however, these long, thin viral particles can be difficult to distinguish by TEM, due to their resemblance to fragments of phage tails and extracellular structures of bacteria (fine pili and flagella).

The use of TEM for estimating rumen viral numbers and population diversity has been largely superseded in favor of metagenomics and molecular methods. Viral genome sequencing can be used to predict viral particle morphology, as specific viral genes are conserved between morphological groups of viruses (Kristensen et al., 2013; Iranzo et al., 2016; Krupovic et al., 2018). However TEM is still widely recognized in virology as an important tool for the detection and morphological characterization of new virus isolates (Cuervo and Carrascosa, 2018; Popov et al., 2019) including those predicted from metagenomics (Shkoporov et al., 2018). The majority of publications to date describing the isolation of phages from the rumen, have used TEM in order to classify new isolates and verify the intact nature of phage particles (Brailsford and Hartman, 1968; Iverson and Millis, 1976a; Styriak et al., 1994; Cheong and Brooker, 2000). Electron microscopy can also be used for visualizing tail fibers involved in host-receptor binding with immunogold labeling, virus:host cell attachment and infection, intracellular viral multiplication and virus particle assembly and release (Ackermann, 1998; Kaelber et al., 2017; Letarov and Kulikov, 2017). TEM and other new, emerging forms of electron microscopy, can therefore be expected to have a continuing, important role in the study of rumen-associated viruses.

### Molecular Biology

In the 1980’s and 1990’s there were rapid technological advancements in the field of molecular biology enabling: the purification of genetic material (DNA and RNA); charge and size-based separation of nucleic acids (electrophoresis; blotting techniques); the use of restriction enzymes for site-specific cutting of DNA and gene mapping; DNA recombination (DNA cloning and genetic transformation); and DNA sequencing techniques (reviewed by Brody and Kern, 2004; Loenen et al., 2014; Heather and Chain, 2016). Rumen microbiologists readily adopted these technologies (Orpin et al., 1988; Flint, 1997) and the way in which rumen phage isolates and rumen viral populations were studied, changed accordingly. Molecular biology was utilized to characterize new phage isolates, with less emphasis on using culture-based methods to understanding phage growth, survival in rumen fluid and host range, and more focus on genome length and restriction enzyme mapping. These analyses were conducted on phage isolates found to specifically infect several strains of rumen bacteria, including *Fusobacterium necroforum* (Tamada et al., 1985), *Selenomonas ruminantium* (Lockington et al., 1988), *Lactobacillus plantarum* (Nemcova et al., 1993), *Bacteroides ruminicola* (Klieve et al., 1991; Gregg et al., 1994), and *Ruminococcus albus* (Klieve et al., 2004). These phage isolates were mainly classified within the viral families Myoviridae and Siphoviridae however, some examples of Podoviridae and Inoviridae were also characterized in this way (Klieve et al., 2004). At this time, whole genome sequencing was technically very challenging and costly and therefore was not routinely undertaken. These studies, however, provided the first insights into the genetic characteristics and unique attributes of the phages infecting rumen bacteria.

Molecular biology also provided new methodologies that could be used for enumerating and describing viral populations occurring within a rumen sample showing a link between populations of rumen bacteria and virus populations (Klieve and Swain, 1993). These methods could also be applied to the analysis of relatively large numbers of rumen fluid samples derived from *in vivo* experiments (Klieve and Gilbert, 2005). The pulsed field gel electrophoresis (PFGE) technique was used following the purification and concentration of virus particles from relatively small volumes (40–60 mL) of rumen fluid and extraction of viral DNA using agarose-embedding techniques, to enable intact genome lengths to be preserved. Separation of DNA by PFGE then produced a population profile, based on viral genome length (Klieve and Swain, 1993). In addition, as an alternative to TEM viral particle counts, a DNA-based method was developed for the estimation of total viral numbers, based on a combination of conventional DNA extraction and electrophoresis techniques, and laser densitometry (Klieve and Swain, 1993). DNA virus population estimates obtained using this technique were slightly higher than those reported using TEM, with estimates of between 3 × 10^9^ to 1.6 × 10^10^ particles per mL of ovine rumen fluid reported (Klieve and Swain, 1993).

Using these techniques (Swain et al., 1996a; Swain, 1999) the rumen viral population was found to (1) have variation in viral populations occurring between individual animals maintained on the same diet, as well as variation between different ruminant species (sheep, goats, cattle); (2) result in blooms of phage activity evidenced by sudden increases or bursts of phage particles; (3) have patterns of diurnal (within day) variation viral populations, in sheep fed once a day, with phage numbers showing blooms of phage replication (lysis events) and similar patterns of variation to those previously observed for bacterial populations in once-a-day fed sheep (Leedle et al., 1982); and (4) be affected by plant compounds found in the ruminant diet (Klieve et al., 1998). For example, the hydrolyzable tannin, tannic acid reduced rumen virus populations when added to sheep prior to feeding to give an intra-ruminal concentration of 0.1% wt/vol., suppressing by as much as 50% the increase in phage numbers normally occurring 8–12 h after feeding (Swain et al., 1996b). The mechanism of this reduction was attributed to the protein-binding activity of the tannic acid, and it was hypothesized that viral particles freely circulating in the rumen were being bound by the tannic acid and removed from the liquid phase (Swain, 1999).

These early molecular biology methods provided information about virus genome length (Hallewell et al., 2014), and enabled estimation of the size and relative length profiles of virus populations (Swain et al., 1996a). However, these methods were unable to deliver any DNA or RNA sequence information about the virus populations observed. Only a few studies conducted at this time were able to provide relatively short, gene-specific sequence information (Gregg et al., 1994). Being able to obtain sufficient amounts of genetic information to be able to infer viral taxonomic classification, was achieved only after significant technological advances in DNA sequencing and the emergence of the field of metagenomics.

### Metagenomic Approaches for Studying Rumen Viruses

Sequencing is an important method for understanding virus populations, and there is a wide variety of tools now at our disposal that makes it increasingly easy to generate virus-specific sequence datasets (referred to as viromes). Metagenomics was initially termed as the study of the collective genomes of all microbial species within an environmental sample (Handelsman et al., 1998) and with the advent of next generation sequencing (NGS), direct sequencing of DNA from environmental samples became possible (Escobar-Zepeda et al., 2015). Microbial community sequencing can be subdivided into two major sequencing methods; (i) shotgun metagenomic sequencing and (ii) amplicon sequencing. Shotgun metagenomic sequencing involves fragmentation of the total environmental DNA for sequencing, while amplicon sequencing includes PCR amplification and sequencing of specific genomic regions of interest. For bacterial taxonomy the 16S rRNA gene is used (McGovern et al., 2018) and can be described as metataxonomics (Marchesi and Ravel, 2015). Whilst the communities of bacteria, archaea, protozoa and fungi can be assessed using both amplicon and shotgun sequencing, the viral component can only be studied using shotgun metagenomics, due to the lack of a universally conserved gene across all viral species (Sullivan et al., 2008; Jameson et al., 2011; Wylie et al., 2015). Shotgun sequencing is highly effective in studying virus populations, which have relatively small genomes (∼20–300 kbp) (Wylie et al., 2015). Whilst DNA based sequencing technologies are most commonly undertaken, this approach excludes the potential study of RNA based viruses, which require total RNA metatranscriptomic sequencing for their detection (Hitch et al., 2019).

The marine environment is an easy system on which to develop viral sequencing tools; it is simple to collect liters of water without negatively impacting the environment being sampled, there is no inhibitory chemistry to prevent sequencing and there is minimal debris and organic matter for viruses to adsorb to and be lost. As a result, marine viral communities were one of the very first environments profiled using metagenomic approaches (Breitbart et al., 2002). Through the use of differential filtration, density-dependent gradient centrifugation, shotgun cloning and sequencing, Breitbart et al. revealed a highly diverse, previously unknown marine viral populations and opened the way for the characterization of viral communities in other environments.

Rumen virology is currently lagging behind marine and even human intestinal virology, with fewer studies using metagenomics to describe viral populations (Table 2). These studies have varied in the procedures used for sample preparation, however, most of the recent studies have included steps for the concentration of viral particles prior to DNA extraction and shot-gun sequencing to generate a viral-specific (virome) dataset. This is in contrast to the early metagenome studies, which generated viromes by extracting virus-only sequences from whole-rumen metagenome datasets (Dinsdale et al., 2008; Willner et al., 2009). As the available sequencing technologies have progressed from Pyrosequencing and Ion Torrent sequencing systems to the more high-throughput Illumina technologies, the rumen virome datasets obtained have increased in sequencing depth and provide more comprehensive sequence coverage. To date the rumen virome of several ruminant species have been examined, including sheep, goats, buffalo and moose, with cattle, including dairy cows and steers being the most frequently investigated (Table 2). Most studies use limited numbers of animals and experimental conditions, such as diet, have remained constant (Berg Miller et al., 2012; Ross et al., 2013).

**TABLE 2:** Summary of published rumen viral metagenomics studies including the type of ruminant used for each study (breed description provided in publication), number of individual animals sampled and the DNA sequencing technologies used.

Ruminant description	Number of animals	DNA Sequencing technology	References
Cattle (Angus Simmental Cross steers)	3	Pyrosequencing	Dinsdale et al., 2008
Cattle (Angus Simmental Cross steers)	3	Pyrosequencing	Willner et al., 2009
Cattle (Holstein dairy cows)	3	Pyrosequencing	Berg Miller et al., 2012
Cattle (Holstein dairy cows)	13	Illumina HiSeq	Ross et al., 2013
Sheep (Cross bred rams)	22	Illumina HiSeq	Yutin et al., 2015
Buffalo (Surti breed)	1	Ion torrent	Parmar et al., 2016
Cattle (steers)	5	Ion torrent, Illumina MiSeq	Anderson et al., 2017
Goats (*Caprus hircus*), sheep (*Ovis aries*)	8, 8	Pyrosequencing	Namonyo et al., 2018
Moose (*Alces alces gigas*)	1	Illumina HiSeq	Solden et al., 2018

Currently only one report has used metagenomics to investigate the effect of changing diets on rumen viral populations which showed the total digestible nutrients was the predominant ecological driver of both bacterial and viral response to dietary change (Anderson et al., 2017). In this study, diversity analysis of both the bacterial and viral communities identified that samples clustered based on diet rather than host, confirming that dietary changes led to consistent changes within both communities (Anderson et al., 2017). This study represented a significant advance in our understanding of rumen viral populations. Diet has been shown in many studies, to greatly influence the relative abundance of rumen microbial species (reviewed by Huws et al., 2018). As viral populations are intrinsically linked to the populations of their microbial hosts (see Lessons to be Learned from Viruses in Other Environments), any changes in the animal diet and consequent availability of specific nutrients for microbial growth in the rumen, can also be expected to cause significance changes the relative abundance of viral populations. While early molecular biology studies eluded to the effects of animal diet on rumen microbial and associated viral populations (Swain et al., 1996a), the advent of viral metagenomics has verified and enhanced our understanding of diet-induced changes on rumen viral populations.

In terms of viral taxonomy the rumen virome varies greatly between individual animals of the same ruminant species and a subset of nearly ubiquitous viral genomic fragments have been identified and proposed to make up a core rumen virome (Anderson et al., 2017). It can also be speculated that these viruses are likely to predate on the bacterial species core to the rumen (Henderson et al., 2015) including the bacterial genera: *Prevotella*, *Butyrivibrio*, *Ruminococcus* and more generally, the families Lachnospiraceae, Ruminococcaceae and Bacteroidales. Metagenomic studies of the rumen have all revealed that the rumen virome is dominated by *Siphoviridae* (32–36%), *Myoviridae* (24–32%), and *Podoviridae* (12–16%), all bacteriophage families belonging to the order Caudovirales (Lefkowitz et al., 2018). As we expect the most abundant rumen viruses to be bacteriophages and public databases are dominated by these, this makes their identification on the basis of genetic homology easier. It is likely that the viral sequencing and identification methods currently adopted, bias against the less common viral groups we anticipate in the rumen, such as archaeal viruses, mycoviruses and other eukaryote viruses.

The dominance of DNA based sequencing methods prevents the study of RNA based viruses including the Totiviridae which infect host-associated protozoa (Grybchuk et al., 2018) and the Partitiviridae, which may exist within the rumen and infect fungi (Hitch et al., 2019). Under these assumptions we may be missing vital functions and relationships. Lower abundance viral families have been identified in the rumen, which have been putatively assigned as large DNA viruses (*Mimiviridae, Phycodnaviridae*), filamentous lysogenic viruses of bacteria and archaea (*Inoviridae*), archaeal viruses (*Bicaudaviridae*) and animal viruses (including; *Baculoviridae*, and *Adenoviridae*) (Berg Miller et al., 2012; Parmar et al., 2016; Anderson et al., 2017). Although they have been detected in rumen virome datasets, the animal viruses identified often represent lineages which would not normally infect rumen microbes. These taxonomic assignments are based on sequence homology to databases of viral sequences available at the time. As both the number of phage genomes in Genbank and publications containing novel viral taxonomies are increasing exponentially, the continuing accumulation of new genetic information, will assist the accuracy of future viral taxonomic classification.

Another application of metagenomics to the study of rumen virus populations involves examining clustered regularly interspaced short palindromic repeats (CRISPR) sequences. The identification of CRISPR-cas arrays and prophages within the cellular component of the rumen microbiome allows for the possible identification of viral-host interactions (Berg Miller et al., 2012). Whilst prophage insertions represent whole viral genomes and are therefore easier to identify and characterize, CRISPR arrays incorporate only a short 25–65 bp region of viral DNA (Shmakov et al., 2017). Despite these short insertions, recent advances have separated and annotated these spacer regions against viral datasets, allowing for viral-host interactions to be identified and studied. Data sets such as the Hungate 1000 rumen collection contain a wealth of data on prophages and viral interactions. The Hungate 1000 collection yielded identifiable CRISPR arrays in over 60% of the isolates (Seshadri et al., 2018). CRISPR arrays can also provide clues to the frequency of aborted lytic viral infections (Sanguino et al., 2015; Edwards et al., 2016). Although these methods have been applied to the rumen microbiome, larger datasets are required to gather enough data for meaningful insights on phage infection (Berg Miller et al., 2012).

Prophages identified from bacterial genomes and microbial metagenome sequencing can help shed light on prophage diversity and the lifestyles of viruses in the rumen. Computational approaches to identify prophages and free viruses in metagenomic data vary depending on the type of data to be analyzed and the prior information available for the virus family of interest. If existing genomic data exists for that viral family, then the direct alignment of reads to the genomes can allow inferences of viral abundances and types in the sample, termed an “aligner” approach (Roux et al., 2015). Another approach, which can utilize reads directly from the sequencer, involves “binning” (separation) of sequences into groups based on characteristics inferred from their sequences. This can be comparing their sequence similarity to known viral sequences (for example Zhao et al., 2017), to more complex approaches which calculate frequency profiles of short strings of DNA, termed “kmers” (for example Willner et al., 2009). This latter approach is based on the observation that different organisms can be identified by the kmer profiles of their genomes and is widely used in metagenomic data analysis (Ren et al., 2017). The resulting “bins” can then be individually assembled into what is hoped to be the genome of a single virus, and these assumptions can be tested by subsequent gene prediction or sequence similarity searches to compare against databases such has those hosted by the NCBI. There is no consensus on which approach or tool is best. The rate at which new tools are emerging (for a current list of tools see Zheng et al., 2019), suggests that using a multi-tool approach may allow balancing of the strengths and weaknesses of each (Anderson et al., 2017). An array of techniques have also been successfully employed on human gut metagenome sequence data to obtain a snapshot of virus communities (Nielsen et al., 2014; Ogilvie and Jones, 2015).

In terms of gene function, the rumen virome has been shown to be relatively consistent across animals of the same ruminant species (Ross et al., 2013). Assigning function to putative viral open reading frames (ORFs) is also difficult, as there is a high percentage of unknown and uncharacterized viral genes (Yooseph et al., 2007). Kyoto Encyclopedia of Genes and Genomes (KEGG) based annotation has suggested that 50–70% of the functional reads within the rumen virome are involved in viral replication (KEGG pathways: nucleotide metabolism, replication and repair), whilst the remaining reads were functionally diverse and included viral structural genes (head and tail proteins). A subset viral genes have been identified as auxiliary metabolic genes (AMGs), which redirect host metabolism toward reactions favorable to phage replication (Anderson et al., 2017). In this way AMGs encompassed in virome datasets represent one way rumen viral populations may influence the metabolic potential of the rumen microbiome.

### Culture-Based Viral Isolations and Genome Sequencing

The first report of culture-based, rumen viral isolation used rumen and non-rumen strains of *Serratia* spp. (facultative anaerobes, family Enterobacteriaceae) to isolate phages, with the intention of demonstrating that phages were prevalent in the rumen of cattle (Adams et al., 1966). As well as successfully isolating phages from rumen fluid, this study also investigated host-specificity (or host range), that is, the ability of these phage isolates to infect multiple strains of bacteria. In this way the study established that phages sourced from the rumen could have limited host-specificity, preferentially infecting *Serratia* host strains sourced from the rumen and being unable to infect non-ruminant *Serratia* strains. Correspondingly, the rumen *Serratia* were not susceptible to infection by *Serratia* phages isolated from soil, water and sewage (Adams et al., 1966). This study was the first to demonstrate that not only were phages prevalent in the rumen, a finding subsequently confirmed using TEM (Hoogenraad et al., 1967), it also demonstrated that the phages found in the rumen differed from those present in other environments.

This primary investigation was followed by numerous studies conducted in laboratories across the globe (Iverson and Millis, 1976a; Tyutikov et al., 1980; Hazlewood et al., 1983; Tamada et al., 1985; Klieve and Bauchop, 1991; Nemcova et al., 1993; Styriak et al., 1994; Ambrozic et al., 2001). The majority of these studies were conducted in the 1980’s and 1990’s, with very few phage isolations reported in the literature beyond this era. To date, most of the viruses isolated from the rumen have been phages infecting rumen bacteria with only a single, preliminary isolation of a rumen-derived archaeal virus being reported (Baresi and Bertani, 1984).

Bacterial hosts strains used for the isolation of rumen phages have been reviewed elsewhere (Gilbert and Klieve, 2015) and include genera classified within various common phyla associated with the rumen, including Firmicutes (*Ruminococcus*, *Lactobacillus*, *Eubacterium*, *Selenomonas*, *Quinella*, and *Streptococcus*), Bacteroidetes (*Bacteroides*), Proteobacteria (*Serratia*), and Actinobacteria (*Bifidobacterium*). The majority of phages infecting rumen bacteria have a typical tailed morphology (Gilbert and Klieve, 2015) and although originally classified according to the Bradley scheme (Bradley, 1967), the following examples are listed according to the modern international committee on taxonomy of viruses (ICTV) classification scheme (King et al., 2012). Rumen phage isolates with Siphovirus morphology include phage F4 which can infect five strains of *Streptococcus bovis* (Styriak et al., 1994; Nigutova et al., 2008); and a long-tailed phage infecting *Prevotella* (*Bacteroides*) *brevis* strain GA33 (Ambrozic et al., 2001). An example of a rumen phage isolate with Myoviridae morphology is phage FnP1 infecting *Fusobacterium necrophorum* strain FnP1 which interestingly also infected 12 other *F. necrophorum* strains of biovars A and B but none of the *Bacteroides* strains tested (*n* = 13) (Tamada et al., 1985). A rumen phage isolate with Podoviridae morphology is phage 2BV which infects *Streptococcus bovis* (Iverson and Millis, 1976a). Reports of phage isolates infecting rumen bacteria with a non-tailed morphotypes have been relatively infrequent, for example, the non-tailed, filamentous Inoviridae phages φRa01 and φRa03 infect *R. albus* AR67 (Klieve et al., 2004).

To isolate phages from the rumen, the majority of culture-based studies have used double-layer agar plates for the detection of clearing zones (plaques) within bacterial monolayers (Klieve, 2005). A combination of anaerobic techniques (Hungate et al., 1964) and rumen fluid-based growth medium with modifications are used to ensure the survival and growth of anaerobic bacterial host strains *in vitro* (Klieve et al., 1989). Culture-based plating techniques have also served as important tools for determining the biological properties of phage isolates, particularly those infecting rumen-associated *Streptococcus* species. Biological properties determined in this way include: viral particle viability in rumen fluid; the development of phage resistance; and host range (Iverson and Millis, 1977; Klieve and Bauchop, 1991; Klieve et al., 1999).

Culture-based plating methods tend to favor the isolation of phages which undergo the lytic cycle of replication. Several different phage replication lifestyles exist in nature and the terminology to describe these lifestyles or cycles, may vary (Hobbs and Abedon, 2016). However, when phages only follow the lytic cycle of replication, phage particle attachment and infection of the host cell leads directly to replication and culminates in the bursting, or lysis, of the host cell to release the progeny phage particles. True lytic phages cannot adopt the alternative, lysogenic lifestyle of replication, as they lack the functional genes required for control of replication and/or integration of phage DNA into the host genome. These phage therefore cannot form a dormant, heritable genetic state (prophage) or establish what is sometimes referred to as chronic infection (reviewed by Weinbauer, 2004; Howard-Varona et al., 2017).

While the isolation of phages from the rumen originally began as a way to determine the extent of viral population diversity, phage isolation was later driven by (1) the need to isolate phages for use in potential biocontrol (phage therapy) of problematic bacteria, such as the bacteria implicated in the development of ruminal acidosis, *Streptococcus bovis* (since re-classified as *S. equinus*) (Tarakanov, 1994); and (2) the requirement for molecular tools (vectors) for the stable transformation of rumen bacteria (Lockington et al., 1988; Gregg et al., 1994). As phage therapy is traditionally based on the use of viable lytic phages, several groups established collections of phages infecting *S. bovis* strains (Iverson and Millis, 1976a; Tarakanov, 1976; Klieve and Bauchop, 1991; Styriak et al., 1994; Klieve et al., 1999). The development of cloning vectors for the genetic transformation of rumen bacteria, however, involved utilizing either phage replication or integration genes, such as those present in lysogenic phages (Gregg et al., 1994; Cheong and Brooker, 1998).

The bacterial genera that were selected for genetic transformation for example *Ruminococcus*, *Bacteroides*, and *Butyrivibrio*, were either those known to be actively involved in the enzymatic breakdown of feed, or examples of common rumen bacteria (Flint, 1997; Wong et al., 2003). Therefore, although many lysogenic phages infecting *S. bovis* were identified (Tarakanov, 1974, 1976; Iverson and Millis, 1976b), only a few phages were genetically characterized (Styriak et al., 2000, 2005). Collectively, however, the numerous isolations of lysogenic phages from the rumen, as well as experiments to chemically induce lysogenic phages from rumen bacteria (Klieve et al., 1989) established, in the absence of DNA sequencing technologies, that a high proportion of rumen bacteria harbor viable lysogenic phages within their genomes.

As DNA sequencing technologies have improved, the whole genome sequencing of bacteria has become more cost-effective and convenient (Didelot et al., 2012). In addition, as bioinformatics methods for genome assembly have become more refined, the detection of integrated prophages has become increasingly possible (Roux et al., 2015; Arndt et al., 2016). Many bacterial and archaeal genomes have been shown to contain fragments or remnants of integrated prophages (Krupovic et al., 2011). For prophage sequences to be considered “intact” (Zhou et al., 2011) and representative of a potentially viable phage, they must contain a full complement of phage genes. These genes are usually clustered into structural and function-related modules including DNA replication and transcriptional regulation, head and tail proteins, DNA and particle packaging, translocation and host cell lysis (Brussow et al., 2004; Roux et al., 2015). While culture-based assessments are required to verify prophage viability, the detection of viral sequences in microbial genome sequences has revolutionized the rate at which novel viruses can be detected.

To date, intact prophages have been detected in the genome sequences of many genera of rumen bacteria (Berg Miller et al., 2012; Gilbert and Klieve, 2015) although the prophage genomes and their homology to known phage sequences, have not been described in detail. Recently prophage sequences detected in the bacterial genome sequences of the ovine rumen isolates *Streptococcus equinus* 2B (∼42 kb, designated φSb2Bpro1) and *Bacteroides ruminicola* ss *brevis* AR29 (∼35 kb, designated φB2bAR29pro1) were described (Gilbert et al., 2017). The latter prophage had been previously examined, with some genetic and biological properties determined, this prophage formed tailed phage particles with a Siphovirus morphology (Klieve et al., 1989; Seet, 2005).

Integrated viral sequences have also been detected in methanogenic archaea isolated from the rumen, including a ∼40 kb prophage of *Methanobrevibacter ruminantium* M1 and a 37 kb prophage of *Methanobacterium formicicum* BRM9 (Attwood et al., 2008; Kelly et al., 2014), although the ability of these prophages to produce intact viral particles has not been reported. The recent international collaborative genome sequencing project, the Hungate 1000 project (Seshadri et al., 2018), analyzed 410 cultured bacterial and archaeal genomes together with their reference genomes and identified virus genes, categorizing these genes according to Pfam numbers (for example pfam03354 Phage Terminase, pfam04860 Phage portal protein and pfam05105 Bacteriophage holin family) and compared them to virus genes found in human intestinal isolates. In this way the study showed that the prophage genes identified differed between the microbes of the herbivore gut and those of the human gut.

Recently whole genome sequences of lytic phages infecting rumen bacterial isolates of *Streptococcus*, *Bacteroides*, and *Ruminococcus* were reported (Gilbert et al., 2017). These phages were of Siphovirus (phages φBrb01 and φBrb02 infecting *B. ruminicola* and phage φSb01 infecting *S. equinus*) and Podovirus morphology (phages φRa02 and φRa04 infecting *R. albus*). Of these phage genomes, the φSb01 sequence had the most genetic homology to other *Streptococcus* phages isolated from non-rumen environments, enabling a more comprehensive annotation of genome and identification of functional modules. In contrast, the Podoviruses φRa02 and φRa04 were more distantly related to known phages, with the few functional genes for which identity could be predicted, all related to genes previously shown to be highly conserved in Podovirus genomes (Iranzo et al., 2016), such as head-tail connector proteins, DNA polymerase and Podovirus encapsidation proteins. Despite this lack of gene annotation, the identified highly conserved phage genes (head-tail connector protein for Podoviridae and phage terminase large subunit (*ter*L) for Siphoviridae) could be employed in phylogenetic analysis to enable the identification of closely related phages. On this basis, φRa02 and φRa04 which infect a *Ruminococcus* host, classified within with the gram positive bacterial phylum Firmicutes, were found to be more highly related to Podoviruses infecting other genera of Firmicutes (for example *Bacillus*, *Clostridium*, and *Streptococcus*), than Podoviruses infecting the gram negative phylum Proteobacteria (for example *Yersinia*, *Pseudomonas*, and *Vibrio*) (Gilbert et al., 2017).

Obtaining the complete genome sequences for rumen virus isolates has many benefits, providing insights into virus:host interactions and facilitating the discovery of novel viral proteins. Most significantly, increasing the number and types of rumen-specific viral genome sequences will greatly enhance the accuracy of viral classification and bioinformatic analysis of viral metagenomics datasets, providing reference sequences and novel viral genes to which meaningful sequence homology can be conferred. Experimental studies with viral isolates is anticipated to remain vital for the determination of important biological properties, such as the determination of virus:host specificity (host range); the characterization of novel viral proteins; determining immunological interactions; and ascertaining viral particle transfer and survival in the gastrointestinal tract and environment.

### Interpreting Virome Function Using Metaproteomics

Metaproteomics is the analysis of the entire protein content of a given microbial community at a specific time point (Wilmes and Bond, 2004). Like many other ‘omic’ technologies, metaproteomics has experienced rapid development in the past 10 years (Kunath et al., 2019), and is quickly becoming commonplace for the analyses of complex microbiome samples (Hagen et al., 2017; Borton et al., 2018; Heyer et al., 2019). Within the rumen microbiome, metaproteomics is a relatively new field, with only sparse datasets generated in recent years (Deusch et al., 2017; Snelling and Wallace, 2017; Hart et al., 2018; Solden et al., 2018). However, multi-omic approaches that combine both metagenome and metaproteome data are now enabling genome-centric resolution that links active metabolic functions to explicit microbial populations in the rumen (Naas et al., 2018; Solden et al., 2018).

Using functional meta-omics to characterize viral taxonomy and function has quickly shown the impact viruses have on ecosystem processes (Emerson et al., 2018). Metaproteomic analysis of viral communities has been used in bioreactors (Heyer et al., 2019) and ocean communities (Brum et al., 2016), which highlighted the presence of newly annotated functions and indications of phage–host interactions causing cell infection. Within the rumen of Alaskan moose, Solden et al. (2018) used a combined meta-omic approach to identify 1,907 viral metagenomics contigs, which clustered into 810 viral populations. This lead to the detection of 148 viral genera, including 35 previously detected from the cow rumen, although the vast majority (75%) were determined to represent previously unknown viral genera.

The moose metaproteome detected a total of 64 viral proteins from 53 different viral contigs, with most proteins (80%) having no known functions, whereas the remainder were largely structural proteins, such as capsids. Against expectations, very few auxiliary metabolic genes (Emerson et al., 2018) were detected, contrary to previous rumen virus findings (Anderson et al., 2017). Examinations of virus–host interaction dynamics, predicted microbial genome hosts, including direct associations between viruses and some of the most active carbohydyrate-degrading and sugar-fermenting populations sampled from the moose rumen (Solden et al., 2018). In particular, numerically dominant *Prevotella*-affiliated populations that play critical roles in hemicellulose degradation were predicted to be infected, highlighting that rumen viruses are likely to be affecting carbon cycling in a top–down manner, whereby changes in the structure and function of the primary fiber degraders may possibly reverberate through the lower trophic levels of the rumen microbiome (Solden et al., 2018). Much more targeted analyses are required to see if such an effect is indeed the case, or if other versatile populations with similar saccharolytic machinery are capable of either supplementing or contributing to, any newly presented niches in such a scenario.

### Expression of Viral Proteins

The study of viral proteins often involves the over-production of the protein of interest *in vitro*. In this way sufficient quantities of protein can be generated to enable analysis using protein chemistry techniques for determination of biological properties. Understanding the biological properties of viral-encoded proteins can lead to an improved understanding of how viruses specifically infect, replicate and interact with host cells and viral proteins have been extensively studied for some well-known, historically important phages for example, the *Escherichia coli* phages lambda (Casjens and Hendrix, 2015) and T4 (Miller et al., 2003); and the Phi29 phages infecting *Bacillus* (Meijer et al., 2001).

The use of viral proteins as new antimicrobials is an area of growing interest, providing an alternative approach to phage therapy, which was traditionally based on the use of whole phage particles to reduce specific microbes (reviewed by Fenton et al., 2010; Abedon, 2012; Shen et al., 2012). Traditionally the use of antibiotics in agriculture was preferred over phage-based therapies for the control of problematic microbes and improving feed efficiency (Russell and Houlihan, 2003). In recent years however, there has been increasing community and regulatory pressure to reduce antibiotic use in agriculture, as well as a drive to reduce enteric methane emissions from ruminant livestock (Hristov et al., 2013; Cameron and McAllister, 2016). One of the antibiotic-free approaches to reduce enteric methane emission harnessed the potential of phage-encoded enzymes to specifically target and lyse rumen methanogens (Klieve and Hegarty, 1999; Leahy et al., 2013). Previously phage-encoded enzymes found in methanogen genomes had only been applied to increase the permeability of methanogen cell walls in order increase the penetration of microscopy stains (Nakamura et al., 2006). Genome sequencing of the rumen methanogen, *M. ruminantium* M1, however, revealed the presence of a prophage gene related to those usually responsible for hydrolyzing the pseudomurein cell walls of methanogens: the endoisopeptidase *pei*R (Attwood et al., 2008) and the potential use of this enzyme was recognized (Leahy et al., 2010).

Recently the *in vitro* expression of *pei*R was reported (Altermann et al., 2018). In this study, initial experiments were conducted to produce this protein using traditional gene cloning, transformation, protein expression in a commercially-available *E. coli* expression strain (Schofield et al., 2015), with the final purified protein employed in lytic activity assays. To further improve the stability and enable practical delivery of the enzyme, an alternative protein expression strategy was adopted. This approach involved the peiR gene being fused to the polyhydroxyalkanoate (PHA) synthase gene which was then expressed in *E. coli* to create PHA bionanoparticles with the lytic enzyme being displayed on the outer surface of the bionanoparticle (Altermann et al., 2018). Using this combination of techniques, sufficient quantities were produced to allow testing for cell-wall degrading effects against several strains of *Methanobrevibacter*, which are usually the most highly abundant methanogen genera found in the rumen (St-Pierre and Wright, 2012), and other methanogenic genera (*Methanobacterium*, *Methanosphaera*, *Methanospirillum*, and *Methanonosarcina*). This study therefore, successfully demonstrated for the first time how viral genes, identified by whole genome sequencing of rumen microbes, can be successfully produced using modern protein expression technologies and developed into a product for activity testing. This study also showed how protein expression techniques are integral to the development of new enzybiotics approaches for the modulation of rumen microbial populations.

### Emerging Technologies

Technologies for studying viruses in environmental samples are constantly being developed, and older technologies are being revisited. New technologies, which could be applied in the context of the rumen, include those being developed to address long-standing biological questions. For example, it is currently difficult to link the extensive populations of viruses identified in viral metagenomes, to their respective microbial hosts (Koskella, 2019). In addition, an older technique, being revisited in the context of the rumen, is the use of Chromatography for size-based virus particle fractionation.

Techniques are evolving that enable us to link viruses to their potential hosts using metagenomic information alone, such as CRISPR-spacer regions (Berg Miller et al., 2012). Hi-C or 3C is another promising technique developed to cross-link DNA in genomes that is distant on the DNA strand, but comes into close physical proximity, due to folding and chromosome architecture (Dekker et al., 2002; Lajoie et al., 2015). This method has the potential to cross-link viral and host DNA during infection to identify viral hosts prior to lysis (Marbouty et al., 2017). Single-cell viral tagging is a further tool that has been tested and proven to work on viruses from the human gut and involves fluorescently labeling phage particles, allowing these to attach to hosts, then sorting and sequencing these pairs (Willner and Hugenholtz, 2013; Džunková et al., 2019).

Chromatography is a traditional chemistry technique for the separation of mixtures, and has the potential be applied for the purification of viruses from rumen samples. Currently the use of chromatography in rumen research has been limited to metabolomic and proteomic studies with only a few publications combining chromatography and virus protein research (Schofield et al., 2015; Altermann et al., 2018). Given the proteinaceous nature of virus structures, particularly phages, implementing protein chromatography methods to separate or isolate whole phages directly should be possible. Despite the size difference and more complicated morphology of phages compared to proteins, it is expected that phages would act like proteins in chromatography systems (Oslizlo et al., 2011). Indeed, chromatography for viral purification is not novel, with literature dating back to 1957, where the coliphages T1, T2r and T2r^+^ were purified from host lysates using ECTEOLA cellulose ion exchange chromatography (Creaser and Taussig, 1957). This emerging technology for separating bacteriophages from environmental samples such as rumen fluid, involves the use of coil column separation and high performance counter current chromatography (HPCCC) (Hu and Pan, 2012; Berthod and Faure, 2015); utilizing the size differences of phages and combining this with the centrifugal forces applied in the HPCCC to separate viruses (Friedersdorff et al., 2018). Although this approach is in its infancy, it is anticipated that depending on the resolution of the CCC technique used, viral fractions based on particle size may be obtained from rumen fluid, enabling the study of less-understood viral morphotypes, for example, viral families which form small, non-tailed icosahedral particles.

## Lessons to Be Learned From Viruses in Other Environments

**FIGURE 2:**
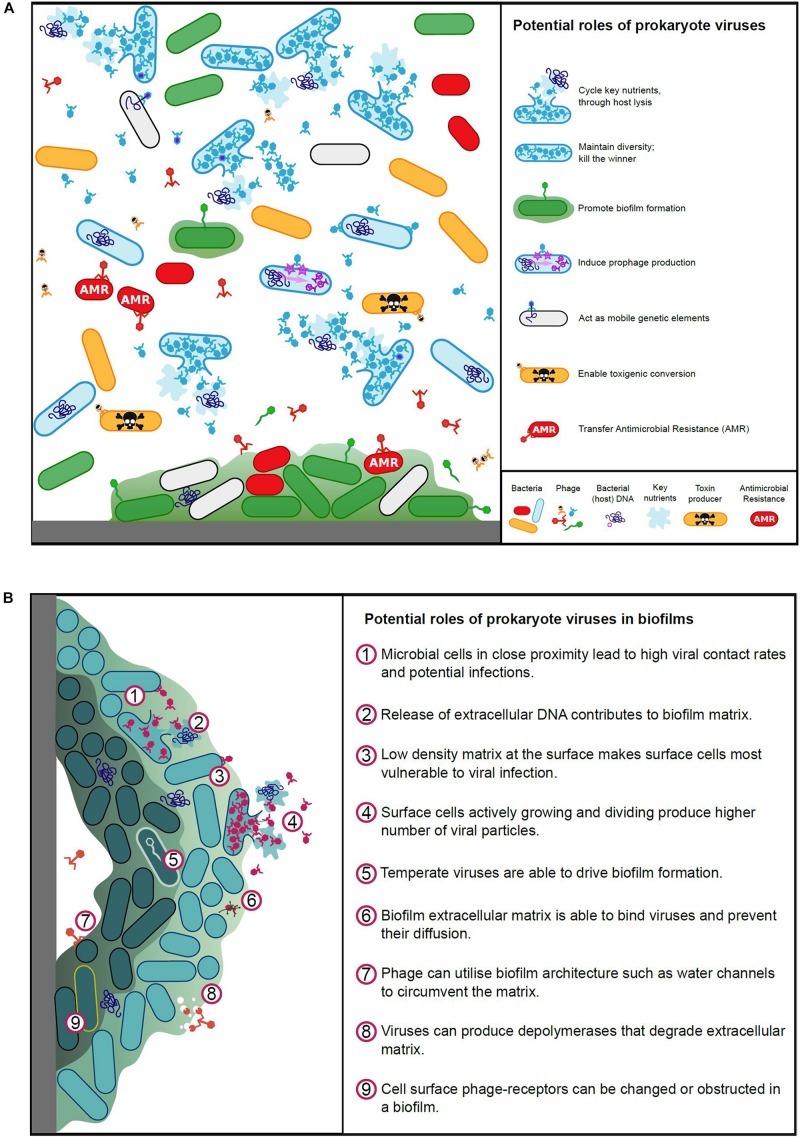
**(A)** Potential roles of prokaryote viruses in microbial ecology and evolution. **(B)** Potential roles of prokaryote viruses in biofilms.

Viruses play a pivotal role in microbial ecology and evolution, contributing to global nutrient cycles, pathogenicity and antimicrobial resistance, and influence animal host immune systems (Salmond and Fineran, 2015). The roles viruses play in aquatic, terrestrial and animal-associated environments are vitally important and viruses, particularly phages, have been acknowledged as being responsible for significant bacterial mortality (Clokie et al., 2011). Our understanding of rumen virus ecology is currently inadequate, we must therefore learn from studies undertaken for other environments, where viruses have been shown to affect microbial populations by maintaining microbial diversity; cycling key nutrients; acting as mobile genetic elements to facilitate genetic exchange; infecting and replicating in a host-specific manner (limited host range); interacting with microbial biofilms (Figure 2) and the immune systems of animal hosts.

### Maintenance of Microbial Diversity

Viruses limit the growth of bacteria in aquatic environments, accounting for an estimated 10–80% of bacterial mortality (reviewed by Weinbauer, 2004). This relentless pressure on bacterial communities shapes their diversity, community structure, function, and temporal dynamics (Letarov and Kulikov, 2009). In the marine environment, the model produced by Thingstad (2000) predicts that viral predation modulates microbial populations via a microbial feedback loop described as the “kill the winner” strategy (i.e., targeting and reducing the size of the most abundant microbial host species). This allows viruses and bacteria to coexist in an equilibrium, thus ensuring microbial diversity. Furthermore, mesocosm experiments by Sandaa et al. (2009) support this model, showing that viruses act as a regulating force, preventing fast growing bacteria from outcompeting slower growers. Viruses have also been shown to control algal blooms (phytoplankton and/or cyanobacteria) occurring in both marine and freshwater environments (reviewed by Weinbauer, 2004).

Within gut-associated microbial communities, viruses are also likely to play a role in modulating microbial populations using predation and microbial feedback loops, particularly during the stages of microbial succession occurring in early life development and at times of microbial community disturbance, e.g., dietary change, dysbiosis and antibiotic use (Lozupone et al., 2012). For example, the “kill the winner” strategy has been observed in the horse gut, where *E. coli* and coliphages co-exist (Golomidova et al., 2007). However, in contrast to the microbial communities found in aquatic microbial ecosystems, the near-theoretical-maximum cell densities combined with the animal immune system lead to temperate phages predominating in animal guts, with the immune system also helping to prevent the over-proliferation of individual gut microbes. In the human gut, phage therapy has been shown to eliminate bacterial pathogens, rather than regulating their numbers, due to the added selective pressure of the immune system (reviewed by Sulakvelidze, 2005).

Microbial hosts have evolved numerous defense mechanisms to prevent phages from killing the winner. These mechanisms target every stage of infection from receptor binding to phage assembly. These include: (1) modification of phage receptors or hosts only display them during part of life cycle; (2) superinfection exclusion, often associated with lysogenic phages to protect their hosts; (3) bacteriophage exclusion (BREX) is a similar host-encoded defense system that prevents phage DNA replication; (4) restriction modification enzymes to degrade foreign DNA, defense island system associated with restriction–modification (DISARM) is more complex, they are found in bacteria and archaea and restricts phage DNA; (5) methylation and CRISPR/*Cas* elements block phage replication (6), abortive infection/programmed cell death (Abi). In addition bacteria can hijack phages to utilize them as gene transfer agents (GTA) and phage-inducible chromosomal islands (PICI) which repackage host DNA into phage structures (for review see Rostøl and Marraffini, 2019).

For each host defense system microbes have evolved, however, viruses appear to have developed a countermeasure, such as the anti-restriction, anti-CRISPR and anti-toxin systems (Dolgin, 2019; Rollie et al., 2020). While these viral countermeasure systems have been largely described for genera not normally found in the rumen, for example, *Pseudomonas* and *Mycobacterium* (Dedrick et al., 2017; Landsberger et al., 2018). Given the extensive microbial and viral diversity observed in the rumen, and the ecological and evolutionary pressures rumen microbial communities experience, these viral countermeasures are likely to exist and play a role in enabling viral replication and persistence in the rumen.

### Cycle Key Nutrients

In aquatic systems viruses have been shown to impact on microbial mortality, causing episodes of microbial lysis, contributing to the recycling of nutrients (Fuhrman and Noble, 1995; Steward et al., 1996; Weinbauer and Höfle, 1998). Viral lysis results in the release of nutrients in the form of dissolved organic matter (DOM) (Shibata et al., 1997). In low nutrient aquatic systems DOM is an essential source of carbon and growth limiting nutrients that is quickly reabsorbed by the microbial community (Bratbak et al., 1990; Proctor and Fuhrman, 1990).

The gut (and rumen) represent much more productive systems than the open ocean, with microbial densities the highest of any studied environment, reaching ∼10^10^–10^11^ mL^–1^ in the rumen (Russell and Hespell, 1981). Due to this high microbial density, these microbes are in constant competition for nutrients and may be subject to control mechanisms, such as virus predation, that maintain stability and cycle nutrients (Lozupone et al., 2012). Due to the microbial competition, nutrients such as iron and vitamin B_12_ (cobalamin), are also limiting to microbial growth in the human gut (Deriu et al., 2013; Degnan et al., 2014).

### Virus-Mediated Genetic Exchange

Viruses infecting microbial populations in various environments have been shown to act as mobile genetic elements, acting as important vectors of horizontal gene transfer and guiding the evolution and diversification of bacteria (Wommack and Colwell, 2000; Koskella and Brockhurst, 2014). Through either generalized transduction (packaging only host bacterial DNA into virus particles) or specialized transduction (mis-packaging host DNA along with virus DNA) viruses contribute to bacterial genetic variability, introducing changes to the genetic make-up of otherwise genetically homogeneous bacterial strains, for example *E. coli* strains causing urinary tract and bloodstream infections (Frost et al., 2005; Ben Zakour et al., 2016). Conversely, the pangenome of dsDNA viruses and prophages infecting a broad range of hosts shows an extensive network of genetic similarity for a subset of viruses, suggestive of a common virus genetic pool and modules of function-related viral genes, resulting in mosaic virus genomes (Hendrix et al., 1999).

Virus genetic exchange from the gut microbiome may have a direct impact on the animal, as well as their bacterial hosts, and virus integrases have been shown to mediate chromosomal integration in human cells (Groth et al., 2000). Lysogenic phages and prophages can influence host phenotype through phage-encoded gene expression, a process termed lysogenic conversion (Brabban et al., 2005). Qin et al. (2010) found that prophages exert a strong influence on the functioning of the human gut microbiome and contribute an astonishing 5% of the conserved microbiome functions observed. Viral DNA can also be incorporated into the genomes of prokaryotes via CRISPR elements: adaptive immune elements that protect against virus infection through targeted nucleases (Barrangou et al., 2007; Koonin et al., 2017). The host-encoded CRISPR spacers can also recombine with incoming virus DNA to perform specialized transduction (Varble et al., 2019).

One of the most studied attributes of viral genetic exchange is their contribution to the development of antibiotic resistance. Antibiotic resistance is a natural bacterial survival strategy, however, the pervasive use of antibiotics in farming and human health-care has led to the development of multidrug-resistant bacterial pathogens (Balcazar, 2014). Antibiotic resistance genes (ARGs) can be subject to virus-mediated horizontal gene transfer (Parsley et al., 2010). Both gram-positive (Ubukata et al., 1975; Mazaheri Nezhad Fard et al., 2011; Goh et al., 2013) and gram-negative (Blahova et al., 1992; Willi et al., 1997; Oliver et al., 2005) bacteria have been shown to carry virus-encoded ARGs. The diversity of ARGs is lower in pathogenic bacteria and environments contaminated with anthropogenic antibiotics, than in unpolluted environments (Muniesa et al., 2013). It has been postulated by Muniesa et al. (2013) that viruses could allow dissemination of these rare ARGs from the natural environment to the human microbiome. A few studies have begun to examine the prevalence and spread of virus-ARGs. In sewage waters, ARGs were detected in virus DNA (Muniesa et al., 2004; Marti et al., 2014). Virus-encoded putative ARGs are also prevalent in healthy human feces, being present in 77% of samples (Minot et al., 2011; Quirós et al., 2014) and have a high carriage rate of in cattle, pigs and poultry (Pereira et al., 1997; Colomer-Lluch et al., 2011).

However, ARGs identified by bioinformatic searches of viral metagenomes of the human and mouse gut may overestimate ARG abundance, as during experimental testing only a few of the predicted ARGs could confer antibiotic resistance *in vitro* (Enault et al., 2017). Experimental testing is vital to determine the functionality of virus-encoded putative ARGs with low sequence identity to bacterial genes. For example, experimental testing has been used to establish the function of virus-encoded β-lactamases identified in the genomes of human gut viruses (Ogilvie et al., 2013). Interestingly, a high proportion of ARGs are carried on plasmids (Bennett, 2008), meaning that even if viral integration-mediated horizontal gene transfer of ARGs is irrelevant, viruses could still facilitate the spread of ARGs, through the release of intact bacterial plasmids following virus-mediated bacterial lysis (Keen et al., 2017).

Another well-studied example of virus-mediated genetic transfer between bacterial genera, is the movement of virus-encoded toxin genes and the evolution of toxin-producing bacteria. A variety of virus-mediated extracellular toxin genes have been identified (listed in Table 3). Conversely, bacteria which develop resistance to phages, rather than allowing the integration of prophage DNA, may become less virulent as a consequence (Leon and Bastias, 2015). In addition to toxicity genes, a number of phage-encoded accessory genes are directly related to pathogenicity and can be expressed alongside toxins. Phage-encoded genes can span an even wider range of functions that increase the fitness of their bacterial hosts including modification of host cell antigens and antibiotic resistance genes (Table 3).

**TABLE 3:** Examples of genes and other factors conveyed by prokaryote viruses which enhance the virulence, pathogenicity and functional capacity of the infected host.

Toxins and factors encoded by prokaryote virus genes	Example publication
Shiga toxin	Smith and Huggins, 1983
Botulinum neurotoxin	Eklund et al., 1971
Diphtheria toxin	Freeman, 1951
Cholera toxin	Abel et al., 2015
Enterohemolysin	Beutin et al., 1993
Cytotoxin	Nakayama et al., 1999
Superantigens	Beres et al., 2002
Leukocidin	Kaneko et al., 1998
Enterotoxin	Betley and Mekalanos, 1985
Scarlet fever toxin	Smoot et al., 2002
Exfoliative toxin	Yamaguchi et al., 2000
Macro-organism invasion	Hynes and Ferretti, 1989
Serum resistance	Barondess and Beckwith, 1990
Phospholipases	Beres et al., 2002
Bacterial adhesion factors	Karaolis et al., 1999
Intracellular survival	Coleman et al., 1989
Antivirulence factors	Ho and Slauch, 2001
Modification of host-cell surface antigens	Wright, 1971
Type III effector proteins	Mirold et al., 1999
Photosynthesis apparatus	Mann et al., 2003
Defense against phage superinfection	Poullain et al., 2008
Antibiotic resistance genes	Balcazar, 2014

### Interactions With the Immune System

In recent years, studies of the human and mouse gastrointestinal tract have provided insights into the establishment of viral populations within the developing gut, and of the interactions occurring between the viruses infecting commensal intestinal microbes and the animal itself. Viruses are rapidly recruited to the human gut within the first few months of life, leading to a diverse viral population dominated by viruses infecting the microbes of the gut, particularly Caudovirales, which then declines in diversity over the following 2 years (Lim et al., 2016). This commensal viral community is an important component of the gut microbiome and plays a role in intestinal inflammation, stimulating a low-level immune response without causing clinical symptoms (Norman et al., 2014).

Growing evidence suggests that viruses have a role in immune defense within the gut mucosa, where they can influence the innate and adaptive immune system (Barr et al., 2015). A number of viruses possess adhesin proteins, with immunoglobulin-like domains, located on the capsid, collar whiskers or tail shaft that are not involved in host attachment, but can facilitate virus attachment to the gut mucosa (Fraser et al., 2007; Barr et al., 2013). Hypervariable regions collocated in either the virus tail fibers and/or immunoglobulin-like genes might help these viruses to evade the adaptive immune system of the gut (Doulatov et al., 2004; Minot et al., 2012). These features allow viruses to attach and persist in the gut mucosa at high concentrations, where they can outnumber bacteria (Barr et al., 2013). Silveira and Rohwer (2016) proposed that the survival strategy employed by virus changes within the mucosa from a lysogenic strategy or “piggyback-the-winner” at the top of the mucosa where hosts are plentiful, to a lytic or “kill-the-winner” strategy deeper in the mucosal layer, where bacterial hosts are scarce.

Although the immune system clears the vast majority of viruses effectively, phages have been shown to be actively transported by transcytosis out of the gut across epithelial cells (Nguyen et al., 2017). This probably accounts for the discovery of phages, delivered to the gastrointestinal tract of mammals for therapy, being found in the blood stream and systemic tissue, where they can trigger both the innate and adaptive immune system (Wolochow et al., 1966; Wolf et al., 1981; Duerr et al., 2004). The translocation of viruses is not always equal among viruses or individuals (Bruttin and Brussow, 2005; Górski et al., 2006).

### Biofilm Formation and Virus: Biofilm Interactions

Biofilms are a community of microorganisms aggregated together within a self-produced matrix (Zobell and Anderson, 1936; Caron, 1987). Biofilms are ubiquitous in nature and most bacteria as well as a number of fungal and yeast species, have the potential to form a biofilm. The biofilms that develop are often poly-microbial and inter-kingdom communities (Swidsinski et al., 2013; Smith et al., 2016; Raghupathi et al., 2018). Within biofilms viruses are often viewed as a predator of bacteria and can therefore offer an alternative to antimicrobial agents against recalcitrant biofilms. The efficacy of viruses against *in vitro* monospecies biofilms has been shown, e.g., in *E. coli* (Doolittle et al., 1995). Not only are viruses are able to prevent biofilm formation (Hibma et al., 1997) and disrupt mature biofilms (Sillankorva et al., 2004; Soni and Nannapaneni, 2010), but they demonstrate synergy with antimicrobial treatments (Sharma et al., 2005; Bedi et al., 2009). The anti-biofilm activity results from not only lysing cells, but also degrading the matrix using depolymerases (Hughes et al., 1998).

Biofilms represent a rich resource for viral production, but the physical nature of biofilms limits the ability of viruses to exploit it (Figure 2B). The biofilm environment brings cells close together and structural elements, such as water channels, allow virus particles rapid access to bacterial cells (Wood et al., 2000; Donlan, 2009). The varying metabolic states within a biofilm, along with the matrix itself however, can pose challenges for viral infection (Doolittle et al., 1996; Hanlon, 2007). To combat this, viruses often produce disruptive enzymes to enable access within the matrix (Hughes et al., 1998; Hanlon et al., 2001). Bacteria at the periphery of a biofilm are most vulnerable to virus adsorption, whilst the dense structure at the center of the biofilm can entrap viruses (Abedon, 2016; Bull et al., 2018; Vidakovic et al., 2018). Hydrophobicity and electrostatic charges are important for viral binding, these can be disrupted within biofilms, and furthermore the aggregation of bacteria may obstruct virus surface receptors (Nordstrom and Forsgren, 1974; Sutherland et al., 2004).

To add to this complexity, viruses has been shown to promote biofilm formation for a number of bacterial genera, such as *Bacillus* and *Actinomyces*, via spontaneous induction of prophage and release of extracellular DNA, which contributes to the extracellular matrix (Schuch and Fischetti, 2009; Carrolo et al., 2010; Wang et al., 2010; Shen et al., 2018). Davies et al. (2016) have also demonstrated that temperate viruses can drive the evolution and adaptation of cells within a biofilm through insertional inactivation of genes. Furthermore, viruses may enhance biofilms through promoting host phenotypic variants and viral particles can physically support the biofilm (Rice et al., 2009). As in any natural system, viruses and biofilms have an ill-defined and complex relationship, with the two entities, being simultaneously at war and peace.

## Roles of Viruses in the Rumen

The role and impact of viruses within the rumen microbial ecosystem has been an area of interest for many years, however, relatively few studies have been conducted to directly address this. Several important aspects of rumen virus populations and the biology of individual viral isolates have been established and on the basis of these findings, the effects of viral activity in the rumen can be inferred (Table 4). Tailed viruses of prokaryotes, classified within the viral order Caudovirales, have been frequently shown to dominate the rumen viral community. These viruses are always present in high numbers, actively replicating in the dense bacterial populations of the rumen and consequently these prokaryote viruses (bacteriophages) are the most studied and best understood.

**TABLE 4:** Roles and characteristics of virus populations within the rumen, as suggested by previous studies of rumen virus populations, rumen microbes (bacteria and archaea) and viruses isolated from the rumen.

Roles and characteristics of virus populations within the rumen	Example publications
Viruses are always present in high numbers and actively replicate in the microbial populations of the rumen.	Adams et al., 1966; Ritchie et al., 1970
Viral replication and action of viral proteins causes lysis of microbial cells and the release of microbial proteins and nucleic acids, contributing to the intra-ruminal recycling of nutrients.	Nolan and Leng, 1972; Orpin and Munn, 1974; Wells and Russell, 1996; Solden et al., 2018
Viruses are mobile genetic elements; they transfer genetic material between microbes and enable the stable integration of virus-encoded (prophage) genes into rumen microbial genomes.	Klieve et al., 1989; Cheong and Brooker, 1998; Attwood et al., 2008; Seshadri et al., 2018
Virus populations drive microbial diversity through predation, causing episodes of microbial lysis and blooms of virus particles.	Hoogenraad et al., 1967; Iverson and Millis, 1977; Klieve and Swain, 1993
Virus infection and development of resistance can cause changes in bacterial growth habit (e.g., increases in bacterial extracellular polysaccharide).	Klieve and Bauchop, 1991; Klieve et al., 1991
Rumen viral population size and composition is dynamic, fluctuating in response to changes in microbial numbers and diet.	Swain et al., 1996a; Klieve et al., 1998; Anderson et al., 2017
Rumen viruses are taxonomically diverse with the prokaryote virus order Caudovirales (tailed phages) being the most abundant.	Ritchie et al., 1970; Klieve and Bauchop, 1988; Berg Miller et al., 2012
Viruses (specifically phages) tend to only infect specific rumen microbes (limited microbial host range).	Iverson and Millis, 1976a; Styriak et al., 1989; Klieve et al., 1999
Virus particles (specifically phage particles) can be degraded with prolonged exposure to rumen fluid.	Iverson and Millis, 1976a; Smith et al., 1987; Nemcova et al., 1993
Virus infection contributes to the development of genetic resistance mechanisms (e.g., CRISPR/*Cas* systems).	Berg Miller et al., 2012; Gilbert et al., 2017
Viruses can interact with cells of the rumen epithelium (demonstrated *in vitro*).	Styriak et al., 1991

Historically, the primary impact of viral populations in the rumen has been their contribution to bacterial cell lysis (Jarvis, 1968; Nolan and Leng, 1972; Swain et al., 1996a). The final stages of viral replication results in the accumulation of intact viral particles inside the cells and fragmentation (lysis) of the cell membrane, causing cell death and the release of progeny viral particles. Lysis also releases remnants of the microbial cell, including cell wall fragments as well as proteins and nucleic acids, which may then be taken up and utilized by other rumen microbes, a process often described as intra-ruminal recycling (Firkins et al., 1992; Hartinger et al., 2018) (Figure 2A). The recycling of nutrients amongst microbes was initially regarded as being a negative consequence of viral activity, limiting the amount of microbial protein carried through to the lower intestine where it can be absorbed by the animal (Leng and Nolan, 1984). A more recent study using proteomics (Solden et al., 2018), suggested that virus-mediated cell lysis also releases intact microbial enzymes, including those involved in carbohydrate breakdown, which in turn facilitates and enhances extracellular feed breakdown within the rumen. This study re-enforces the initial hypotheses that virus populations significantly impact on microbial lysis and nutrient recycling within the rumen, but in contrast, has also provided an alternative, more positive perspective on the long standing question of whether viral-mediated microbial lysis is detrimental or beneficial to rumen function.

The second major impact of rumen viral populations is that they modulate microbial populations, notably dominant bacterial populations, causing viral blooms. These viral blooms were first observed in studies monitoring phage population numbers over a 24 h period in once-a-day fed sheep (Swain et al., 1996a). As virus particles, even those sourced from the rumen, are degraded by rumen fluid (Orpin and Munn, 1974; Tarakanov, 1976) there must be constant viral replication to replenish and sustain viral numbers. Blooms of lytic activity may reflect top–down or “kill the winner” activity, with dominant microbial populations being targets of viral predation, however this has never been shown experimentally for the rumen. Given the functional redundancy of rumen microbial populations, whereby multiple microbial species can perform the same function (Henderson et al., 2015; Weimer, 2015), it can be anticipated that blooms of lytic phage activity are unlikely to impact on overall rumen efficiency, with the niche made available by the selective removal of dominant genera, being rapidly filled by a bacterial population with similar fermentative capacity. This turn-over of microbial populations, however, represents the way viruses can drive and maintain microbial diversity in the rumen.

The third proposed impact of rumen viral populations is their capacity to act as mobile genetic elements (MGE). In this way, viruses can pick up and transfer genetic material between the microbes which they infect and replicate in. The most notable examples include transducing phages, which readily package the host DNA into phage particles (Brussow et al., 2004), and lysogenic phages which form a stable heritable state with the host cell (Weinbauer, 2004; Touchon et al., 2017). Of these, lysogenic phages infecting rumen bacteria and archaea have been most frequently reported (see section “Culture-Based Viral Isolations and Genome Sequencing”). It has also been suggested in a bioinformatics study that lysogenic phages can transfer auxiliary metabolic genes (AMGs) between rumen bacteria, resulting in metabolic re-programming (Anderson et al., 2017). In other microbial environments virus-mediated genetic transfer also contributes to the development of antimicrobial resistance and toxigenic conversion (see section “Virus-Mediated Genetic Exchange”).

Although several rumen genera have been shown to produce antimicrobial proteins, for example bacteriocins produced by *Streptococcus* and *Ruminococcus* (Chen and Weimer, 2001; Azevedo et al., 2015), there have been no investigations into the extent of toxin production by rumen microbes and the contribution of viruses to toxigenic conversion is not understood. Rumen microbes have been shown to demonstrate antimicrobial resistance (Mantovani and Russell, 2001; Benahmed et al., 2014) and the use of antibiotics in feed supplements can contribute to the development of this resistance (Russell and Houlihan, 2003; Cameron and McAllister, 2016). The contribution of viruses to the development of antimicrobial resistance in the rumen has not been investigated. Most of the more abundant rumen microbial genera are not recognized as important zoonotic pathogens and if virus-mediated toxigenic conversion does occur, given the lack of published data, it appears to have had little impact on ruminant or human health. Instead the lower intestine of ruminants represents more of a public health concern, because the intestine can harbor zoonotic and highly pathogenic *E. coli* strains, particularly strains that have undergone toxigenic conversion following infection by Shiga-toxigenic phages (Brussow et al., 2004; Bonanno et al., 2016).

**FIGURE 3:**
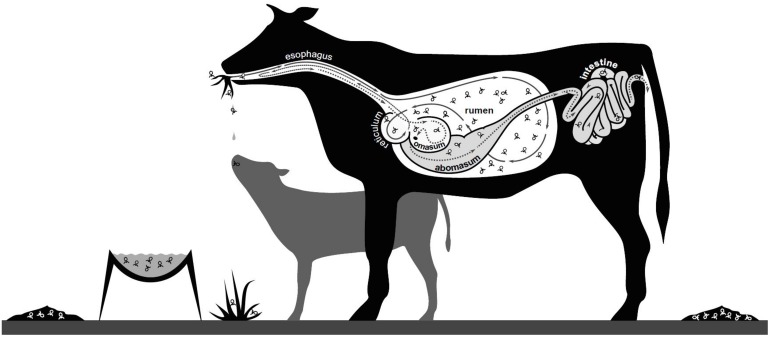
Potential mechanisms for spread of rumen viruses. Viruses may be spread by aerosols, saliva and fecal shedding, with viral particles moving between animals (maternal and within herd interactions) and their environment (water, feed, housing and infrastructure).

The other potential impacts of viruses on microbial populations are in accordance with observations of viral populations in other environments, but have not been extensively investigated. These impacts include: (1) Virus activity facilitating the formation of biofilms, with bacteria changing their physical growth habit, forming clumps and layers of extracellular polysaccharide in response to phage infection and the development of phage resistance (Klieve and Bauchop, 1991). (2) The ruminant gastrointestinal tract may never be free of viruses as commensal microbes and their associated viruses may simultaneously colonize the ruminant gut during early life development. In addition, viruses may be readily introduced into the rumen with between-animal transfer of viruses occurring via aerosols and saliva from close contact (e.g., maternal transfer, especially pre-weaning) and from shared feed and water sources, as well as housing and farm infrastructure (Figure 3). (3) Viruses infecting rumen bacteria may interact with the cells of the rumen wall, eliciting an immune response. For all these potential roles, further investigations and technological advances are required, e.g., to link specific virus and host populations, to provide both experimental evidence and new insights, into how rumen viral populations contribute to the rumen microbiome, fermentation efficiency and overall ruminant growth and condition.

## Future Directions and Applications

Our ability to explore microbial communities has expanded with our ability to sequence microbes quickly and cheaply from any given environment. This presents an amazing opportunity to investigate viruses without the requirement to grow them, or their host. Techniques are evolving that enable us to link viruses to their potential hosts using metagenomic information alone; such as matching CRISPR-spacer regions in hosts to homologous sequences in their viruses which also provides clues to the frequency of viral interactions (Berg Miller et al., 2012; Sanguino et al., 2015; Edwards et al., 2016), or, through linking physically close genomic information by proximity ligation, such as infected host genomes and viral progeny prior to release (Marbouty et al., 2017). Our ability to interpret metagenomic sequence data gives us a tantalizing insight into virus-host community dynamics, but a deeper understanding of functionality is hindered by a lack of basic knowledge of the roles and functions of the organisms in the rumen. There is a dearth of basic knowledge of rumen viruses and that is critical to understanding their function; such as prophage induction, auxiliary genes and functional roles, lytic virus virulence, replication rate, host range and burst size. The future of rumen viral work will need to focus on two areas: answering outstanding questions (Table 5) and developing practical applications.

**TABLE 5:** Future rumen virus research: outstanding questions for further investigation.

Outstanding questions
Do viruses infect all of the microbial populations found in the rumen?
How do virus populations impact on biofilm formation, colonization of plant material and rumen fermentation?
To what extent do virus populations impact on microbial lysis and nutrient recycling within the rumen?
What are the biological properties of rumen viruses (e.g., virus:host interactions, host range, replication strategies, virus particle survival and persistence in the environment)?
Do viruses impact on microbial gene transfer in the rumen and what types of genes may they transfer?
Do rumen virus communities change from early life to mature animals and can they change throughout the lifetime of an animal?
Do viruses from the rumen interact with the rumen wall and GI tract and cause immunological effects in the animal?
Can phage therapy (lytic viruses or viral enzymes) be used to remove problematic organisms (e.g., methanogens) from the rumen?

To address the outstanding questions, rumen viral research will need to be carried out on three fronts, standard laboratory based *in vitro* work, coupled with *in vivo* rumen studies to inform *in silico* computer modeling which will generate testable hypotheses. Advances in sequencing and proteomics provide vast quantities of data that enable us to characterize, understand and model the role of rumen viruses. Basic knowledge of the rumen microbiome as a whole, including viruses, is essential to allow us to explore the rumen microbiome through modeling (Louca and Doebeli, 2017). Modeling of whole microbial communities opens the possibility to understand community functionality and dynamics (Widder et al., 2016). Rumen modeling has largely concentrated on the impacts of nutrition on emissions and efficiency rather than on the microbes and their functions (Ellis et al., 2009; Mills et al., 2014; Gregorini et al., 2015). A recent study examined a simple rumen microbiome consisting of representatives of three microbial groups: Bacteroidetes, Firmicutes, and Archaea, plus their associated viruses (Islam et al., 2019). This simple model seeks to identify novel metabolic interplay between these microbes, to demonstrate metabolite exchange and the potential importance of viral-encoded auxiliary metabolic genes. Modeling is one tool to understand rumen function and opens the door to explore the use of viruses as alternatives to antibiotics to increase feed efficiency, maintain robust animal health and lower methane production.

The current widespread use of antibiotics as feed additives can be viewed as unsustainable (Economou and Gousia, 2015) and phage therapy shows promise as a targeted approach to control bacterial populations and promote animal health. Natural viruses have been used through phage therapy to remove problematic bacteria, archaea and their biofilms, from plants, animals and food crops, to humans, aquaculture and poultry (Donlan, 2009; Monk et al., 2010; Sarker et al., 2016). More recently engineered viruses have been used clinically to treat human patients with multi-antibiotic resistant infections (Dedrick et al., 2019), however this has been on a compassionate basis, as phage therapy is not currently authorized as a medicinal product. Temperate viruses that integrate into their host genomes also have the potential to confer positive genetic traits, but tend to be avoided due to the negative traits they can also confer (Monteiro et al., 2019). To develop effective phage therapies, we still need to generate rumen-specific information on viral dose size and viral particle longevity, to overcome potential viral inactivation by factors such as tannins, antibodies, macrophages, and clearance rates, as well as undertake basic viral characterization of adsorption efficiency, latency and burst size (Ly-Chatain, 2014). Phage therapy has been applied to ruminants with varying results (Gill et al., 2006; Johnson et al., 2008), however, this has been aimed primarily at the lower intestine (Dini et al., 2012) and has yet to be widely exploited in the rumen.

Phage therapy using intact viruses is highly host specific and presents regulatory issues, the use of virus-derived products or “enzybiotics” could directly replace conventional antimicrobials (O’Mahony et al., 2011; Shen et al., 2012; Louca and Doebeli, 2017). Mechanisms for the delivery and release of viral material from the host often involves enzymes (lysins/holins, peptidases, depolymerases, tail spike proteins), which can be repurposed as enzybiotics (Murphy et al., 1995; Andres et al., 2012; São-José, 2018). Indeed, enzybiotics have been tested in animals (O’Flaherty et al., 2009) and targeted nanoparticles displaying enzybiotics have been demonstrated to reduce methanogenesis by rumen-associated archaea (Altermann et al., 2018). Virus-derived enzybiotics are appealing, due to their scalability and ease of use within existing guidelines, enzybiotics could represent the future direction of phage research.

## Conclusion

The rumen microbial community is integral in maintaining ruminant health, feed efficiency and is responsible for methanogenesis. Viruses are numerous, ubiquitous components of microbial communities, including those found in the rumen and have been overlooked in the past due to the technical difficulties experienced in their study. From the moment viral particles were first visualized it was clear that rumen viruses deserved investigating. However, direct study was limited to observations and indirect study proved problematic. This has led to rumen viruses being either ignored or overlooked in favor of more rewarding studies. Our current understanding shows that rumen viruses are not only numerically important, but are rapidly turned over. Within the rumen microbiome viruses enable nutrient cycling, genetic exchange and appear to promote functional stability. We can also extrapolate from other environments their likely influence on the rumen, but these require experimental testing.

Fortunately, new technologies are emerging that allow the scientific community to answer many of the fundamental questions pertaining to viruses; what viruses are present, how abundant are they and how do viruses interact with microbes, influence microbial community function and what impact do rumen microbial viruses have on the whole animal? Whole microbiome modeling, metagenome sequencing and metaproteomics are powerful and exciting tools that will enable us to answer these questions and test our predictions. Viruses offer the chance to understand, model and manipulate complex rumen communities. The future potential of viral applications are many and varied and require a more integrative research effort. Viruses are an intrinsic part of rumen microbial communities and are no more or less important than any other component. It is vital that viruses be incorporated into well-designed, large-scale studies that encompass all components of the rumen microbiome for both fundamental and hypothesis driven research. With affordable, powerful tools at our disposal we have a fantastic opportunity to explore rumen viruses and provide practical applications, based on sound knowledge of the whole ruminant.

## Author Contributions

RG and EJ conceived and designed the manuscript. RG and EJ wrote the majority of the manuscript with ET, TH, JF, CC, and PP contributing specialist sections to the manuscript. EJ, ET, RG, and KC contributed to the figures and tables. RG prepared the rumen viral fraction. KC undertook the electron microscopy. DO critically reviewed the manuscript and contributed to the table improvement. All authors approved the final version of the manuscript.

## Conflict of Interest

The authors declare that the research was conducted in the absence of any commercial or financial relationships that could be construed as a potential conflict of interest. The reviewer, SN, declared a shared affiliation, with no collaboration, with several of the authors, RG and DO, to the handling editor at the time of review.
